# Timing of nasogastric tube placement after endovascular thrombectomy and risk of stroke-associated pneumonia: a retrospective cohort study

**DOI:** 10.3389/fneur.2026.1845093

**Published:** 2026-06-12

**Authors:** Xiao-Fei Hu, Bai-Ling Pan, Li Han

**Affiliations:** 1 Department of Neurology, Taizhou Hospital of Zhejiang Province, Affiliated to Wenzhou Medical University, Linhai, Zhejiang, China; 2 Department of Endocrinology, Taizhou Hospital of Zhejiang Province, Affiliated to Wenzhou Medical University, Linhai, Zhejiang, China

**Keywords:** acute ischemic stroke, endovascular thrombectomy, nasogastric tube, pneumonia risk, stroke-associated pneumonia

## Abstract

**Background:**

Stroke-associated pneumonia (SAP) is a common complication following endovascular thrombectomy (EVT), yet the impact of nasogastric tube (NGT) placement timing on SAP risk has not been examined.

**Methods:**

We conducted a single-center retrospective cohort study of 331 patients who underwent successful EVT and received NGT placement between June 2022 and May 2025. The primary exposure was time from reperfusion to NGT placement. Multivariable logistic regression and restricted cubic splines were used to examine the association between NGT timing and SAP, adjusting for age, sex, admission NIHSS score, serum albumin, hypertension, atrial fibrillation, and diabetes mellitus.

**Results:**

Stroke-associated pneumonia occurred in 227 patients (68.6%), reflecting the cohort’s restriction to EVT patients requiring NGT placement, a high-aspiration-risk subgroup. Each 12-h delay in NGT placement was associated with a 33% increase in the adjusted odds of SAP (aOR 1.33, 95% CI 1.06–1.68, *p* = 0.015). Patients with NGT placement more than 8 h after reperfusion had significantly higher odds of SAP than those with earlier placement (aOR 1.73, 95% CI 1.04–2.90, *p* = 0.036). Restricted cubic spline analysis demonstrated a monotonically increasing dose–response relationship (*P* overall = 0.073). The association appeared stronger in patients without atrial fibrillation (aOR 2.38) than in those with atrial fibrillation (aOR 1.08).

**Conclusion:**

In EVT-treated patients requiring NGT placement, longer time to NGT insertion was associated with higher SAP risk after adjustment for measured covariates. These findings suggest that NGT placement timing may be a potentially modifiable factor in post-EVT care, and provide a hypothesis-generating basis for prospective evaluation.

## Introduction

Acute ischemic stroke remains a leading cause of mortality and long-term disability worldwide, accounting for a substantial proportion of the global burden of neurological disease (1). Endovascular thrombectomy (EVT) has markedly changed the prognosis of large vessel occlusion stroke, with successful recanalization now achievable in over 80% of treated patients (2). Despite this, a considerable proportion of patients who achieve reperfusion fail to attain good functional outcomes, in part because of complications arising in the early post-procedural period (3–5).

Stroke-associated pneumonia is among the most frequent and consequential of these complications. Across stroke populations, SAP affects approximately 10 to 45.8% of patients, with substantially higher rates reported in those treated with endovascular thrombectomy (6–12). In EVT-treated patients, the combination of severe neurological deficits, procedural sedation, and post-reperfusion hemodynamic instability creates a particularly vulnerable physiological state (3–5). SAP is independently associated with increased in-hospital mortality, prolonged hospitalization, and worse functional outcomes at 90 days (13, 14). Multiple mechanisms contribute to SAP in this setting, including aspiration of oropharyngeal secretions, stroke-induced immunodepression, and loss of protective airway reflexes (7, 15, 16).

Nasogastric tube placement (NGT) is a standard component of care for patients with severe stroke who cannot safely swallow (17, 18). By providing a dedicated route for enteral nutrition and medication delivery, NGT bypasses the oropharyngeal phase of swallowing, reduces the frequency of aspiration events, and supports early nutritional repletion. Although early enteral nutrition has been associated with improved outcomes in critically ill patients more broadly, the question of when NGT should be placed after EVT has not been systematically examined. In clinical practice, NGT placement is often deferred during the acute post-procedural monitoring window, with timing determined by individual physician judgment, nursing availability, and patient tolerance rather than by evidence-based protocols. Whether this delay carries clinical consequences for SAP risk has not previously been quantified.

In this study, we examined the association between the timing of NGT placement after successful reperfusion and the risk of SAP in a cohort of 331 patients treated with EVT at a single center. We hypothesized that longer intervals between reperfusion and NGT placement would be associated with higher odds of SAP, and we sought to identify a pragmatic timing threshold to inform the design of future prospective studies.

## Methods

### Study design and participants

This was a single-center retrospective cohort study conducted at Taizhou Hospital of Zhejiang Province, affiliated with Wenzhou Medical University, China. We screened consecutive patients with acute ischemic stroke who underwent endovascular thrombectomy (EVT) between June 2022 and May 2025. Eligible patients were those who achieved successful vascular recanalization, defined as a modified Thrombolysis in Cerebral Infarction (mTICI) grade of 2b or 3 (19), and who received nasogastric tube (NGT) placement during hospitalization. We excluded patients with unsuccessful recanalization (mTICI <2b; *n* = 45), those hospitalized for fewer than 72 h owing to early death or self-discharge (*n* = 53), those with pre-existing infection or antibiotic exposure within 2 weeks before EVT (*n* = 23), and those who did not undergo NGT placement (*n* = 293). A total of 331 patients were included in the final analysis. The study was approved by the Institutional Review Board of Taizhou Hospital (approval number: K20260121), and the requirement for individual informed consent was waived given the retrospective nature of the study. Data were extracted from electronic medical records, procedural logs, and laboratory information systems by the first author using a standardized data extraction form developed for this study.

### Exposure: NGT placement timing

The primary exposure was the time interval from successful reperfusion to NGT placement, measured in hours and recorded from procedural and nursing records. This variable was analyzed both as a continuous measure and dichotomized at 8 h. The 8-h threshold was identified through two complementary approaches: a multivariable-adjusted grid search across candidate cutoffs from 4 to 24 h in 1-h increments, which identified 8 h as the value maximizing the association with SAP; and receiver operating characteristic (ROC) analysis, in which the Youden index was highest at 7.7 h, supporting 8 h as the clinically interpretable threshold (area under the curve 0.562, *p* = 0.035).

### Outcome: stroke-associated pneumonia

The primary outcome was stroke-associated pneumonia (SAP), defined using modified Centers for Disease Control and Prevention (CDC) criteria as new-onset pneumonia occurring within 7 days after successful reperfusion (20). Diagnosis required the presence of a new or progressive infiltrate on chest imaging together with at least two of the following clinical features: fever exceeding 38 °C, increased sputum production, new or worsening cough, and oxygen desaturation. SAP diagnoses were made by the treating clinicians during routine clinical care, applying the modified CDC criteria above. For the present analysis, the first author re-reviewed each case against the full medical record (clinical notes, chest imaging reports, and microbiological data) using a structured form to confirm that the diagnostic criteria were met. Because both the exposure and outcome data were retrieved from the same medical records, outcome ascertainment could not be performed blinded to NGT placement timing.

### Covariates

Candidate covariates were selected *a priori* based on clinical plausibility and existing literature on SAP risk. These included age, sex, admission National Institutes of Health Stroke Scale (NIHSS) score, vascular risk factors (hypertension, diabetes mellitus, atrial fibrillation), procedural characteristics (intravenous thrombolysis, general anesthesia, procedure duration, TICI grade), and laboratory values obtained within 24 h after the procedure (serum albumin, blood glucose, platelet count, creatinine, fibrinogen, hemoglobin). Post-procedural serum albumin was included as a surrogate for nutritional status, given its established relevance to infection susceptibility and aspiration risk in stroke patients (21).

### Statistical analysis

Continuous variables are presented as median [interquartile range] and categorical variables as number (percentage). Between-group comparisons used the Mann–Whitney *U* test for continuous variables and the chi-squared or Fisher exact test for categorical variables, as appropriate.

We constructed three logistic regression models of increasing covariate adjustment to examine the association between NGT placement timing and SAP. Model 1 was unadjusted. Model 2 adjusted for age and sex. Model 3 additionally adjusted for admission NIHSS score, post-procedural serum albumin, hypertension, atrial fibrillation, and diabetes mellitus. These covariates were selected on the basis of *a priori* clinical reasoning and were not subject to data-driven variable selection. Inflammatory markers measured after EVT (WBC, neutrophil count, NLR) were not included in the outcome model, as these post-procedural values may reflect early infection rather than baseline confounding, and their inclusion could introduce collider bias. All variables included in the primary analysis were complete for the 331 patients; complete-case analysis was therefore performed. Multicollinearity among Model 3 covariates was assessed using variance inflation factors; all values were below 1.24, well below the conventional threshold of 5, indicating no problematic collinearity. The exposure was modeled both as a continuous variable (per additional hour, per 6 h, and per 12 h to aid interpretability) and dichotomously (NGT placement >8 h versus ≤8 h after reperfusion).

To characterize the dose–response relationship between placement timing and SAP risk without imposing a linear assumption, we fitted restricted cubic splines (RCS) with three knots placed at the 5th, 50th, and 95th percentiles of the exposure distribution, using the 10th percentile as the reference value. Pointwise 95% confidence intervals were obtained by bootstrapping (500 replications). Overall and nonlinear associations were assessed using likelihood ratio tests from the multivariable RCS model (Model 3 covariates). A sensitivity analysis comparing 3-knot, 4-knot, and 5-knot specifications and a linear model was performed to confirm the choice of specification; the 3-knot model was selected as the best-fitting spline specification.

To address potential confounding by indication, we performed an inverse probability of treatment weighting (IPTW) sensitivity analysis. Stabilized weights were derived from a propensity score model including age, sex, admission NIHSS, post-procedural albumin, hypertension, atrial fibrillation, and diabetes mellitus, and used to weight a logistic regression model for SAP on NGT timing (per 12-h delay). A doubly robust estimator combining these weights with outcome regression on the same covariates was also computed. Covariate balance was assessed by standardized mean differences before and after weighting.

Subgroup analyses were performed across six pre-specified clinical strata: age (<70 versus ≥70 years), sex, hypertension, diabetes mellitus, atrial fibrillation, and admission NIHSS score (<15 versus ≥15). For each subgroup, the association between NGT placement timing (>8 h versus ≤8 h) and SAP was estimated using the same logistic regression specification as Model 3, omitting the stratifying variable from the covariate set. Effect modification was evaluated by including a multiplicative interaction term between NGT timing and the subgroup variable. All six subgroups were pre-specified before analysis on the basis of established SAP risk factors; the corresponding interaction tests should be regarded as exploratory given the limited number of events in some strata.

A two-sided *p*-value below 0.05 was considered statistically significant. All analyses were performed using R statistical software (version 4.5.0; R Foundation for Statistical Computing, Vienna, Austria).

## Results

### Patient characteristics

**Figure 1:**
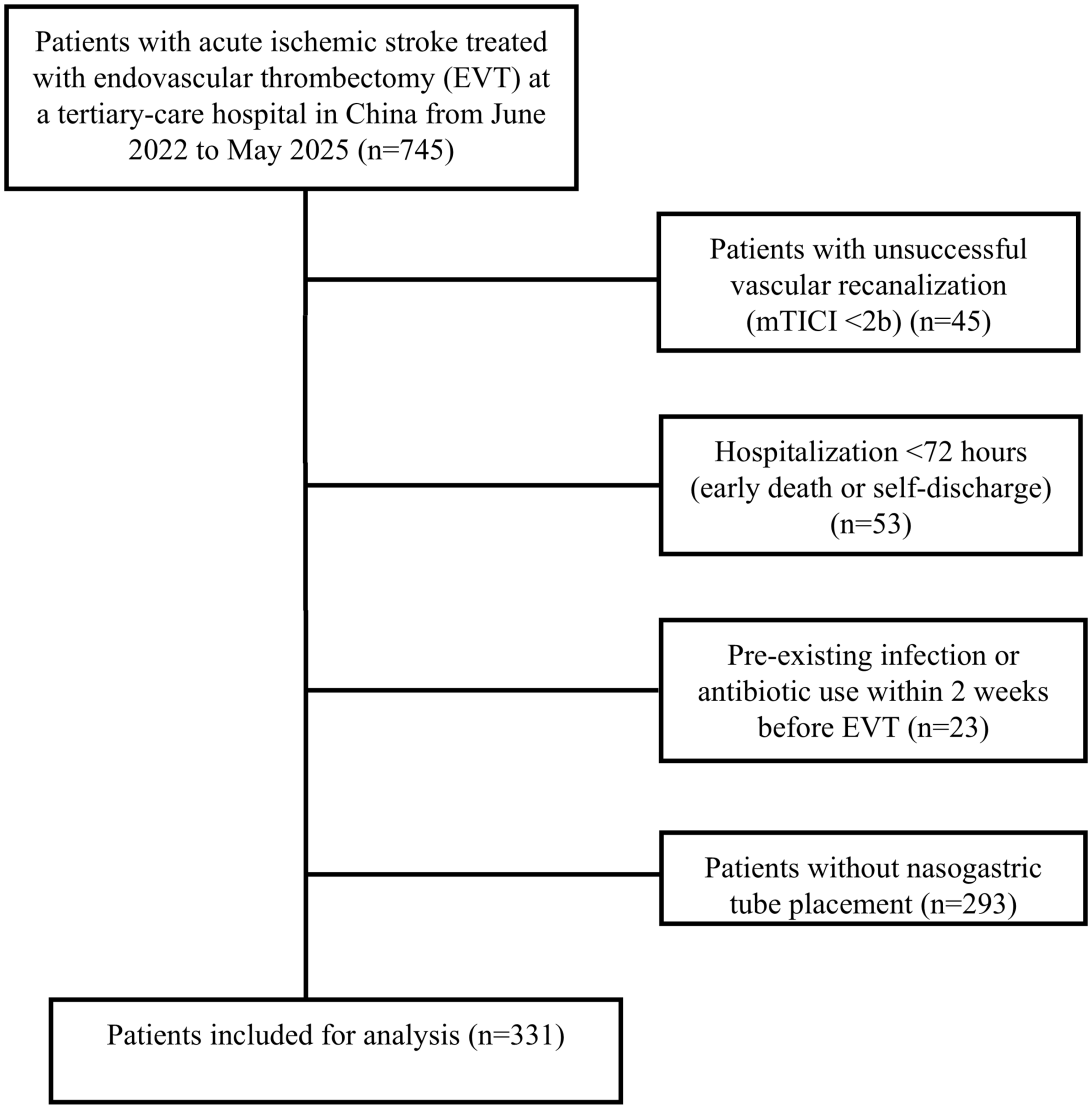
Study flow diagram. Of 745 consecutive patients with acute ischemic stroke who underwent endovascular thrombectomy (EVT) at Taizhou Hospital of Zhejiang Province between June 2022 and May 2025, 331 were included in the final analysis after applying the following exclusion criteria: unsuccessful vascular recanalization (modified Thrombolysis in Cerebral Infarction [mTICI] grade <2b; *n* = 45), hospitalization of fewer than 72 h due to early death or self-discharge (*n* = 53), pre-existing infection or antibiotic use within 2 weeks before EVT (*n* = 23), and absence of nasogastric tube placement during hospitalization (*n* = 293).

Of 745 patients who underwent EVT at our center between June 2022 and May 2025, 331 met the inclusion criteria and formed the analytic cohort (Figure 1). The median age was 74.0 years [IQR 66.0, 80.0], and 136 patients (41.1%) were female. Atrial fibrillation was present in 168 patients (50.8%) and hypertension in 218 (65.9%). The median admission NIHSS score was 16.0 [11.0, 19.0], reflecting a predominantly severe stroke population. Intravenous thrombolysis was administered before EVT in 79 patients (23.9%), and general anesthesia was used in 22 (6.6%). The median puncture-to-reperfusion time was 65.0 min [45.0, 101.5].

The SAP occurred in 227 patients (68.6%). Compared with those who did not develop SAP, patients with SAP were older (74.0 [68.0, 81.0] vs. 70.0 [60.8, 78.0] years, *p* < 0.001), had higher admission NIHSS scores (17.0 [12.0, 21.0] vs. 13.0 [9.0, 17.0], *p* < 0.001), and more frequently had hypertension (70.5% vs. 55.8%, *p* = 0.013). Post-procedural serum albumin was lower in the SAP group (35.8 vs. 37.3 g/L, *p* = 0.012); conversely, NLR (7.7 vs. 6.5, *p* = 0.014) and neutrophil count (7.2 vs. 6.7 × 10^9^/L, *p* = 0.030) were higher. Sex, atrial fibrillation, diabetes, and procedural variables did not differ significantly between groups. Full baseline characteristics are presented in Table 1.

**Table 1:** Baseline characteristics of patients stratified by stroke-associated pneumonia.

Variables	Overall (*n* = 331)	Non-SAP (*n* = 104)	SAP (*n* = 227)	*p*-value
Demographics				
Age, median [IQR], years	74.0 [66.0, 80.0]	70.0 [60.8, 78.0]	74.0 [68.0, 81.0]	<0.001
Female sex, *n* (%)	136 (41.1)	46 (44.2)	90 (39.6)	0.505
Vascular risk factors				
Hypertension, *n* (%)	218 (65.9)	58 (55.8)	160 (70.5)	0.013
Diabetes mellitus, *n* (%)	76 (23.0)	18 (17.3)	58 (25.6)	0.130
Atrial fibrillation, *n* (%)	168 (50.8)	45 (43.3)	123 (54.2)	0.084
Prior stroke/TIA, *n* (%)	84 (25.4)	20 (19.2)	64 (28.2)	0.109
Coronary artery disease, *n* (%)	24 (7.3)	8 (7.7)	16 (7.0)	1.000
Admission clinical assessment				
NIHSS on admission, median [IQR]	16.0 [11.0, 19.0]	13.0 [9.0, 17.0]	17.0 [12.0, 21.0]	<0.001
Systolic BP, median [IQR], mmHg	151.0 [132.0, 167.0]	150.0 [131.8, 164.0]	152.0 [132.0, 168.5]	0.219
Diastolic BP, median [IQR], mmHg	85.0 [75.5, 96.5]	87.0 [76.0, 96.0]	83.0 [75.0, 97.0]	0.717
Heart rate, median [IQR], bpm	78.0 [68.0, 92.0]	77.0 [65.0, 89.0]	79.0 [70.0, 93.0]	0.133
Body temperature, median [IQR], °C	36.5 [36.2, 36.8]	36.5 [36.3, 36.8]	36.6 [36.2, 36.8]	0.875
Procedural characteristics				
IVT, *n* (%)	79 (23.9)	22 (21.2)	57 (25.1)	0.519
General anesthesia, *n* (%)	22 (6.6)	4 (3.8)	18 (7.9)	0.251
PRT, median [IQR], min	65.0 [45.0, 101.5]	69.0 [47.8, 98.0]	64.0 [42.0, 102.0]	0.331
TICI grade 3, *n* (%)	261 (78.9)	80 (76.9)	181 (79.7)	0.662
Post-EVT laboratory values (within 24 h)				
Albumin, median [IQR], g/L	36.0 [33.7, 38.7]	37.3 [34.6, 39.8]	35.8 [33.5, 38.4]	0.012
Blood glucose, median [IQR], mmol/L	7.2 [6.3, 8.8]	6.9 [6.1, 8.2]	7.3 [6.3, 9.0]	0.079
Platelet, median [IQR], ×10^9^/L	180.0 [145.0, 221.5]	187.0 [152.8, 225.2]	178.0 [142.5, 219.5]	0.154
Creatinine, median [IQR], μmol/L	68.0 [57.0, 85.5]	65.0 [55.8, 79.2]	69.0 [57.5, 86.5]	0.071
Fibrinogen, median [IQR], g/L	3.0 [2.6, 3.6]	3.0 [2.6, 3.6]	3.0 [2.6, 3.5]	0.709
Hemoglobin, median [IQR], g/L	124.0 [112.0, 134.0]	126.5 [113.0, 136.8]	123.0 [112.0, 131.0]	0.082
WBC, median [IQR], ×10^9^/L	8.4 [6.6, 10.3]	7.9 [6.4, 9.6]	8.6 [6.7, 10.7]	0.048
Neutrophil count, median [IQR], ×10^9^/L	6.9 [5.3, 8.7]	6.7 [4.7, 8.0]	7.2 [5.3, 9.1]	0.030
Lymphocyte count, median [IQR], ×10^9^/L	0.9 [0.7, 1.3]	1.0 [0.8, 1.4]	0.9 [0.7, 1.2]	0.110
NLR, median [IQR]	7.0 [4.9, 10.9]	6.5 [4.3, 8.9]	7.7 [5.2, 11.2]	0.014
NGT placement				
Time to NGT placement, median [IQR], h	7.2 [1.6, 18.1]	3.8 [1.4, 15.4]	9.3 [1.6, 19.2]	0.070
NGT placement >8 h, *n* (%)	159 (48.0)	39 (37.5)	120 (52.9)	0.013

SAP, stroke-associated pneumonia; NIHSS, National Institutes of Health Stroke Scale; BP, blood pressure; IVT, intravenous thrombolysis; PRT, puncture-to-reperfusion time; TICI, Thrombolysis in Cerebral Infarction; WBC, white blood cell count; NLR, neutrophil-to-lymphocyte ratio; NGT, nasogastric tube; IQR, interquartile range.

The median time from reperfusion to NGT placement was 7.2 h [1.6, 18.1] overall. NGT was placed within 8 h of reperfusion in 172 patients (52.0%) and after 8 h in 159 patients (48.0%). The SAP rate was higher in those with delayed placement (75.5% vs. 62.2%; absolute difference 13.3 percentage points, *p* = 0.013).

### Association between NGT placement timing and SAP

In unadjusted analysis, each additional hour of delay in NGT placement was associated with a 2% higher odds of SAP (OR 1.02, 95% CI 1.00 to 1.04, *p* = 0.031). This association persisted after adjustment for age and sex (Model 2: OR 1.02, 95% CI 1.00 to 1.04, *p* = 0.022) and remained consistent in the fully adjusted model incorporating admission NIHSS, serum albumin, hypertension, atrial fibrillation, and diabetes mellitus (Model 3: OR 1.02, 95% CI 1.00 to 1.04, *p* = 0.015). When expressed per clinically meaningful time intervals, each additional 6 h of delay corresponded to an adjusted OR of 1.15 (95% CI 1.03 to 1.29), and each additional 12 h to an adjusted OR of 1.33 (95% CI 1.06 to 1.68).

Using the 8-h threshold, patients with NGT placement more than 8 h after reperfusion had significantly higher odds of SAP compared with those with earlier placement in all three models (Model 1: OR 1.87, 95% CI 1.16 to 3.01, *p* = 0.010; Model 2: OR 1.83, 95% CI 1.13 to 2.99, *p* = 0.015; Model 3: OR 1.73, 95% CI 1.04 to 2.90, *p* = 0.036). These findings are summarized in Table 2.

**Table 2:** Association between NGT placement timing and SAP.

Variable	Model 1		Model 2		Model 3	
	OR (95% CI)	*P*	OR (95% CI)	*P*	OR (95% CI)	*P*
NGT timing as continuous variable						
Per 1-h increase	1.02 (1.00–1.04)	0.031	1.02 (1.00–1.04)	0.022	1.02 (1.00–1.04)	0.015
Per 6-h increase	1.13 (1.01–1.26)	0.031	1.14 (1.02–1.27)	0.022	1.15 (1.03–1.29)	0.015
Per 12-h increase	1.27 (1.02–1.58)	0.031	1.30 (1.04–1.62)	0.022	1.33 (1.06–1.68)	0.015
NGT timing as binary variable (ref: ≤8 h)						
>8 h vs. ≤8 h	1.87 (1.16–3.01)	0.010	1.83 (1.13–2.99)	0.015	1.73 (1.04–2.90)	0.036

Model 1: Crude (unadjusted).

Model 2: Adjusted for age and sex.

Model 3: Adjusted for age, sex, NIHSS on admission, albumin, hypertension, atrial fibrillation, and diabetes mellitus.

SAP incidence: Overall 227/331 (68.6%); NGT ≤8 h group 107/172 (62.2%), median NGT time 1.6 [1.0, 3.0] h; NGT >8 h group 120/159 (75.5%), median NGT time 18.4 [13.9, 25.1] h.

NGT, nasogastric tube; SAP, stroke-associated pneumonia; OR, odds ratio; CI, confidence interval; IQR, interquartile range; NIHSS, National Institutes of Health Stroke Scale.

### Dose–response analysis using restricted cubic splines

**Figure 2:**
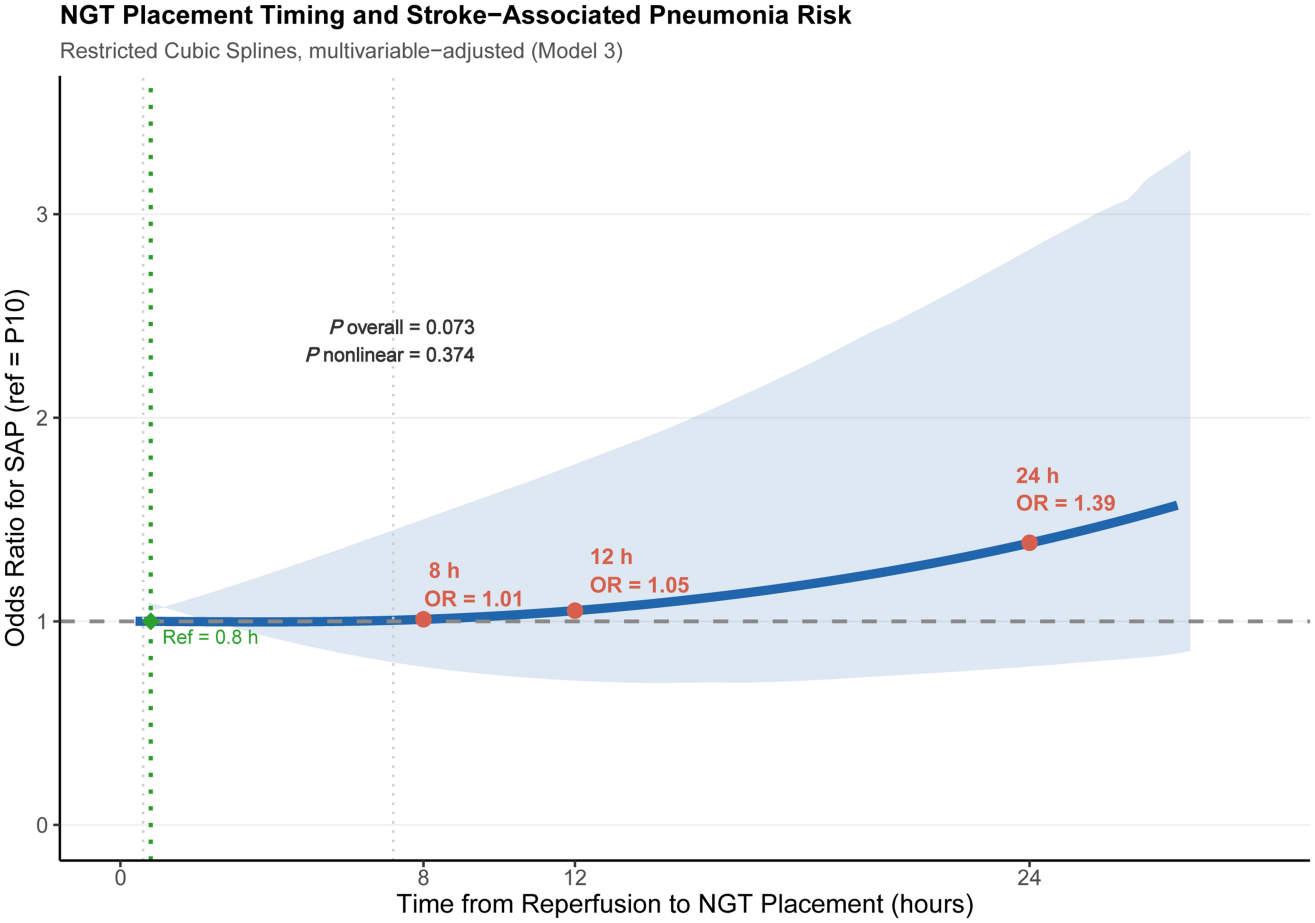
Dose–response relationship between nasogastric tube placement timing and stroke-associated pneumonia risk. Restricted cubic spline analysis of the association between time from reperfusion to nasogastric tube (NGT) placement and the odds of stroke-associated pneumonia (SAP), with knots placed at the 5th, 50th, and 95th percentiles of the NGT timing distribution (0.6, 7.2, and 42.8 h, respectively). The reference value is set at the 10th percentile (0.8 h). The solid line represents the multivariable-adjusted odds ratio and the shaded area represents the 95% confidence interval, derived from 500 bootstrap iterations. Selected time points are annotated: 8 h (aOR 1.01), 12 h (aOR 1.05), and 24 h (aOR 1.39). The horizontal dashed line indicates an odds ratio of 1.0. Vertical dotted lines indicate the spline knot positions. The model is adjusted for age, sex, admission National Institutes of Health Stroke Scale score, serum albumin, hypertension, atrial fibrillation, and diabetes mellitus. *P* overall = 0.073; *P* non-linear = 0.374. NGT, nasogastric tube; SAP, stroke-associated pneumonia; aOR, adjusted odds ratio; CI, confidence interval.

To evaluate the shape of the association without imposing a linear constraint, we modeled the relationship between NGT placement timing and SAP risk using restricted cubic splines with knots at the 5th, 50th, and 95th percentiles of the timing distribution (0.6, 7.2, and 42.8 h, respectively). The overall association was of borderline significance (*P* overall = 0.073), with no evidence of a nonlinear relationship (*P* non-linear = 0.374). Across the observed range of placement times, the adjusted OR increased monotonically from approximately 1.01 at 8 h to 1.05 at 12 h and 1.39 at 24 h, relative to the reference value of 0.8 h (10th percentile). The spline curve and its 95% confidence interval are shown in Figure 2.

### Sensitivity analyses for confounding by indication

The propensity score model showed that none of the seven baseline covariates significantly predicted NGT placement timing (McFadden pseudo *R*^2^ = 0.012; Supplementary Table S1), suggesting that timing variation in this cohort was not strongly driven by recorded clinical characteristics. Stabilized weighting achieved excellent covariate balance (maximum absolute SMD = 0.013; Supplementary Table S2). Sensitivity estimates were consistent with the primary analysis: the stabilized IPTW estimator yielded aOR 1.20 per 12-h delay (95% CI 1.01–1.43; *p* = 0.038), and the doubly robust estimator yielded aOR 1.33 (95% CI 1.09–1.63; *p* = 0.006), identical in magnitude to Model 3.

### Subgroup analyses

**Figure 3:**
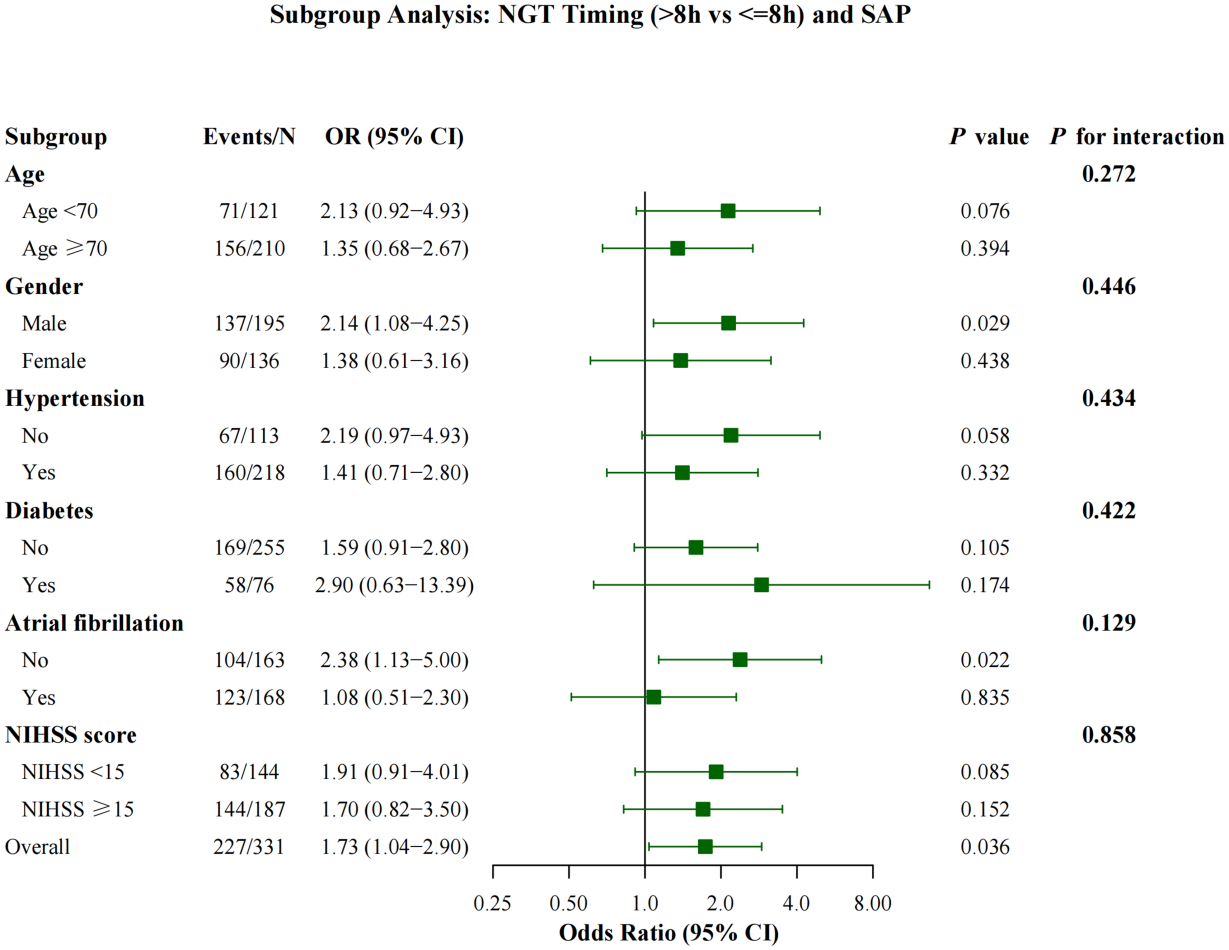
Subgroup analysis of the association between nasogastric tube placement timing and stroke-associated pneumonia. Forest plot showing the association between NGT placement more than 8 h versus 8 h or less after reperfusion and SAP risk across six pre-specified clinical subgroups: age, sex, hypertension, diabetes mellitus, atrial fibrillation, and admission NIHSS score. Each subgroup estimate was derived from a multivariable logistic regression model with the same covariate adjustment as Model 3, excluding the stratifying variable. The overall estimate (aOR 1.73, 95% CI 1.04–2.90, *p* = 0.036) is shown at the bottom. *P* for interaction was assessed by including a multiplicative interaction term between NGT timing and the subgroup variable in the full model. Square markers represent point estimates and horizontal lines represent 95% confidence intervals. Marker size is proportional to the number of events in each subgroup. NGT, nasogastric tube; SAP, stroke-associated pneumonia; OR, odds ratio; CI, confidence interval; NIHSS, National Institutes of Health Stroke Scale; AF, atrial fibrillation.

Pre-specified subgroup analyses were conducted across six clinical strata (Figure 3). The association between delayed NGT placement and SAP was directionally consistent across all subgroups. No statistically significant interaction was detected for age (*p* = 0.272), sex (*p* = 0.446), hypertension (*p* = 0.434), diabetes (*p* = 0.422), or admission NIHSS score (*p* = 0.858).

Two patterns of note emerged from the subgroup analysis. First, the association was significant among patients without atrial fibrillation (aOR 2.38, 95% CI 1.13 to 5.00, *p* = 0.022) but not among those with atrial fibrillation (aOR 1.08, 95% CI 0.51 to 2.30, *p* = 0.835; *P* for interaction = 0.129). Second, a directionally similar gradient was seen by stroke severity: the effect estimate was numerically larger in patients with admission NIHSS below 15 (aOR 1.91, 95% CI 0.91 to 4.01) than in those with NIHSS of 15 or above (aOR 1.70, 95% CI 0.82 to 3.50).

## Discussion

In this retrospective cohort of 331 patients undergoing successful EVT for acute ischemic stroke, delayed NGT placement was associated with a higher risk of SAP after adjustment for measured covariates. Each 12-h increment in placement time corresponded to a 33% increase in the adjusted odds of SAP, and patients who received NGT more than 8 h after reperfusion had approximately 70% higher odds than those with earlier placement. The dose–response relationship was also examined using restricted cubic spline analysis, which showed a monotonically increasing trend across the observed range of placement times. We are not aware of prior studies specifically examining the timing of NGT placement in relation to SAP risk in patients undergoing EVT.

The SAP rate of 68.6% in our cohort is higher than published rates in unselected EVT populations (typically 25–55%) (9–12), and is explained by the cohort’s composition: all included patients received NGT placement, which itself reflects clinically significant dysphagia, impaired consciousness, or perceived aspiration risk. The cohort therefore represents the more neurologically compromised end of the EVT population (median admission NIHSS 16, median age 74), among whom high SAP rates are expected regardless of NGT timing. Within this high-risk group, the relevant question is whether placement timing, rather than placement itself, influences SAP risk. The findings should accordingly be interpreted as applying specifically to EVT-treated patients in whom NGT placement is clinically indicated, and external validity to the broader EVT population requires further investigation.

Several pathophysiological mechanisms link delayed enteral access to SAP. Dysphagia occurs in up to 65% of acute stroke patients and is a major determinant of aspiration pneumonia (7). In EVT-treated patients, high NIHSS scores, frequent use of general anesthesia, and the hemodynamic perturbations of procedural sedation compound the baseline impairment in protective airway reflexes (7, 12). In the interval between reperfusion and NGT placement, patients are dependent on oral or parenteral nutrition, with repeated swallowing attempts exposing the compromised airway to aspiration (7). Early NGT placement bypasses the oropharynx, redirecting nutrition delivery and reducing the frequency and volume of aspiration events (17).

Beyond the mechanical protection afforded by enteral feeding, NGT placement may also attenuate the immune suppression that follows acute stroke (15, 16). Stroke-induced immune depression syndrome, mediated through sympathetic and hypothalamic–pituitary–adrenal axis activation, renders patients vulnerable to bacterial infections within the first 24–72 h after ictus (15, 16). Early enteral nutrition maintains gut barrier integrity, limits bacterial translocation, and preserves mucosal immune function, each of which may limit the progression from colonization to established pneumonia (22, 23). Whether the observed association between NGT timing and SAP is mediated through aspiration reduction, immune modulation, or both cannot be determined from the present data.

The 8-h threshold identified in this study reflects a point at which the cumulative risk of aspiration and immune vulnerability may become clinically significant. This cutoff was derived through a multivariable grid search and consistent with ROC analysis, in which the Youden-optimal cutoff was 7.7 h, providing some empirical support for this exploratory threshold, which requires prospective validation before it can be considered a clinical target. In practice, 8 h aligns with a natural window in EVT care: the acute post-procedural monitoring period, during which NGT placement may be deferred pending neurological reassessment or logistical constraints. Our findings suggest that once this window has elapsed, the risk of SAP may begin to rise. Whether earlier placement confers additional benefit relative to placement between 4 and 8 h cannot be resolved from this analysis given the limited power of finer subgroup comparisons, and this question remains open.

The subgroup analysis revealed two patterns that, considered together, may suggest a shared biological explanation, though the evidence remains preliminary. The association between delayed NGT placement and SAP was evident in patients without atrial fibrillation (aOR 2.38, 95% CI 1.13 to 5.00, *p* = 0.022) but not in those with atrial fibrillation (aOR 1.08, 95% CI 0.51 to 2.30, *p* = 0.835), with a non-significant interaction term (*p* = 0.129). A directionally similar pattern was observed by stroke severity: the association was numerically stronger in patients with admission NIHSS below 15 (aOR 1.91, 95% CI 0.91 to 4.01) than in those with NIHSS of 15 or above (aOR 1.70, 95% CI 0.82 to 3.50), though neither subgroup reached statistical significance independently. These subgroup findings are exploratory and should not be interpreted as evidence of differential benefit by AF status or stroke severity.

Both observations are broadly consistent with stroke severity as a shared explanatory factor. In patients with atrial fibrillation, the greater ischemic burden associated with cardioembolic large vessel occlusion may independently drive SAP risk to a degree that attenuates the relative contribution of NGT timing (24, 25). However, the interaction term for AF was not statistically significant (*p* = 0.129), and the subgroup analyses are underpowered; these findings are exploratory and should not be interpreted as evidence of differential benefit by AF status.

This study has several limitations that bear on the interpretation of the findings. Although we adjusted for major clinical covariates and performed IPTW and doubly robust sensitivity analyses, residual confounding from unmeasured factors remains an important limitation of observational studies of this kind. Bedside dysphagia screening, level of consciousness, and individual physician judgment regarding NGT placement were not systematically captured, and these are likely among the most important determinants of both NGT timing and SAP risk. The retrospective, single-center design further limits causal inference. The principal SAP diagnostic criteria are largely objective (radiological infiltrate, fever, oxygen desaturation), which limits the scope for subjective ascertainment bias, but residual measurement error cannot be excluded. The indication for NGT placement and the precise clinical trigger for timing were not systematically recorded, which prevents us from fully disentangling whether delayed placement reflects a deliberate clinical decision or logistical constraints. Because both the exposure and outcome data were retrieved from the same medical records, outcome ascertainment could not be performed blinded to NGT placement timing; this is an inherent limitation of the retrospective design. The study was conducted at a single center in China, and the findings may not generalize to healthcare settings with different procedural workflows or patient populations. Finally, the exclusion of 293 patients who did not receive NGT placement represents a substantial proportion of the EVT cohort; these patients may have had systematically different baseline characteristics and SAP rates. The findings therefore apply specifically to EVT-treated patients in whom NGT was clinically indicated and should not be extrapolated to the broader EVT population. Data extraction was performed by a single reviewer, and formal inter-rater reliability assessment was not conducted; this is a minor methodological limitation.

### Clinical implications

Despite these limitations, the findings carry potential clinical relevance. NGT placement timing is a potentially modifiable care process, and the 8-h post-reperfusion threshold identified here provides a candidate target for prospective evaluation rather than an immediately actionable quality-improvement standard. Prospective studies and, ultimately, randomized evaluation of early versus deferred NGT placement in EVT-treated patients are needed to establish whether the association observed here reflects a causal relationship and to define which subgroups are most likely to benefit.

## Conclusion

In this single-center retrospective cohort of EVT-treated patients, delayed NGT placement was associated with increased SAP risk after adjustment for measured covariates, with an 8-h post-reperfusion threshold identified as an exploratory boundary that requires prospective validation. These findings suggest that NGT placement timing may warrant further investigation as a potential target in post-EVT care. Prospective evaluation is warranted to establish causality and to identify which patient subgroups derive the greatest benefit from early enteral access.

## Data Availability

The raw data supporting the conclusions of this article will be made available by the authors, without undue reservation.
