# Supplementary material for: Timing of nasogastric tube placement after endovascular thrombectomy and risk of stroke-associated pneumonia: a retrospective cohort study
Source: Front Neurol. 2026 Jun 12;17:1845093. doi: 10.3389/fneur.2026.1845093 (PMC13303366; doi:10.3389/fneur.2026.1845093)
Supplement: Supplementary file 1 [file Table_1.DOCX]

**Supplementary Table S1. Propensity score model used to derive stabilized weights for the IPTW sensitivity analysis**

| **Variable** | **OR (95% CI)** | **β coefficient** | ***P* value** |
| --- | --- | --- | --- |
| Age (per year) | 1.01 (0.99–1.03) | +0.012 | 0.304 |
| Female sex | 0.87 (0.55–1.38) | −0.137 | 0.560 |
| Admission NIHSS | 1.01 (0.98–1.05) | +0.014 | 0.358 |
| Albumin (g/L) | 0.97 (0.92–1.01) | −0.032 | 0.179 |
| Hypertension | 1.03 (0.64–1.66) | +0.027 | 0.913 |
| Atrial fibrillation | 0.79 (0.49–1.27) | −0.235 | 0.328 |
| Diabetes mellitus | 1.22 (0.71–2.09) | +0.197 | 0.473 |

*Logistic regression model for the binary indicator of NGT placement >8 hours after reperfusion. Stabilized weights derived from this model were applied to the continuous exposure (per 12-hour delay) in the IPTW sensitivity analysis reported in the response letter. McFadden pseudo R² = 0.012; likelihood ratio χ² = 5.53, df = 7, p = 0.596. None of the seven baseline covariates significantly predicted NGT placement timing, indicating that timing variation in this cohort was not strongly driven by recorded baseline clinical characteristics. OR, odds ratio; CI, confidence interval; NIHSS, National Institutes of Health Stroke Scale.*

**Supplementary Table S2. Covariate balance before and after stabilized inverse probability of treatment weighting**

| **Variable** | **Unweighted** |  |  | **Weighted** |  |  |
| --- | --- | --- | --- | --- | --- | --- |
|  | **≤8 h** | **>8 h** | **SMD** | **≤8 h** | **>8 h** | **SMD** |
| Age (years) | 71.86 | 73.04 | +0.107 | 72.42 | 72.46 | +0.003 |
| Female sex (%) | 41.9 | 40.3 | −0.033 | 41.5 | 41.4 | −0.002 |
| NIHSS | 15.68 | 16.37 | +0.097 | 16.01 | 16.02 | +0.001 |
| Albumin (g/L) | 37.06 | 36.21 | −0.175 | 36.72 | 36.78 | +0.012 |
| Hypertension (%) | 65.1 | 66.7 | +0.033 | 65.8 | 66.0 | +0.002 |
| Atrial fibril. (%) | 52.3 | 49.1 | −0.065 | 50.8 | 50.7 | −0.002 |
| Diabetes (%) | 22.1 | 25.2 | +0.072 | 23.6 | 23.7 | +0.002 |

*Standardized mean differences (SMDs) of the seven baseline covariates between groups defined by the binary cutoff (NGT placement >8 h vs ≤8 h), before and after stabilized inverse probability of treatment weighting. Weights derived from the propensity score model in Supplementary Table S1 were applied in the IPTW sensitivity analysis of the continuous exposure (per 12-hour delay). All SMDs were below the conventional threshold of 0.10 after weighting (maximum |SMD| = 0.012 for albumin), indicating excellent balance. Weight range: 0.74–1.70. No participants required trimming, reflecting strong overlap of propensity score distributions between the two groups. SMD, standardized mean difference.*
